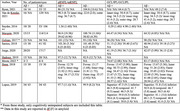# Association between retinal changes assessed by OCT and cerebral amyloid burden measured by PET imaging in Alzheimer's continuum: a systematic review

**DOI:** 10.1002/alz70856_097082

**Published:** 2025-12-24

**Authors:** Sara KamaliZonouzi, Mobina Amanollahi, Mahsa Dolatshahi, Melina Farshbafnadi, Sara Samadzadeh

**Affiliations:** ^1^ Tehran University of Medical Sciences, Tehran, Tehran, Iran (Islamic Republic of); ^2^ Mallinckrodt Institute of Radiology, Washington University in St. Louis, St. Louis, MO, USA; ^3^ Universität zu Berlin, Berlin, Brandenburg, Germany

## Abstract

**Background:**

Optical coherence tomography (OCT) is known to be an effective tool for the diagnosis of ocular degenerative diseases like glaucoma. Its application in cerebral neurodegenerative diseases is mainly studied due to its cost‐effectiveness compared to other modalities like positron emission tomography (PET); however, its utility is yet to be elucidated. This systematic review investigates the association of the extent of retinal degeneration, quantified by OCT, with cerebral amyloid beta (Aβ) burden measured by PET.

**Method:**

PubMed, Embase, Scopus, and Web of Science were systematically searched (November 11, 2024) to identify studies that examined the association between OCT and brain Aβ burden within the AD spectrum and cognitively normal (CN) individuals. Two authors independently performed the screening, data extraction, and synthesis processes. Extracted data encompassed participant demographics, OCT metrics—specifically macular retinal nerve fiber layer (mRNFL), peripapillary RNFL (pRNFL), and ganglion cell‐inner plexiform layer (GCIPL) thicknesses—and cerebral Aβ burden as measured by PET imaging.

**Result:**

Eleven eligible studies with 221 Aβ+ subjects and 535 Aβ‐ subjects were included in this systematic review, which analyzed retinal changes across CN, preclinical AD, and cognitively impaired individuals, categorized as Aβ+ and Aβ‐. The most used PET tracers were 11C‐PiB PET and 18F‐florbetaben. Two studies reported a significant reduction in RNFL in association with Aβ pathology, while seven studies reported non‐significant alterations. Four studies reported a significant reduction in GCIPL in association with Aβ pathology, while six others reported non‐significant alterations in both.

**Conclusion:**

Overall, while the exact association between retinal changes and brain Aβ‐ status remains inconclusive, this review suggests that retinal changes revealed by OCT, especially GCL/IPL reduction, could be assistive in the early detection of AD and Aβ positivity. However, further studies employing standardized methodologies and well‐defined participant subgroups are needed to validate its potential for clinical use.